# Editorial: Phagocytosis: Molecular Mechanisms and Physiological Implications

**DOI:** 10.3389/fimmu.2020.586918

**Published:** 2020-09-11

**Authors:** Esther M. Lafuente, Florence Niedergang, Carlos Rosales

**Affiliations:** ^1^Department of Immunology, Ophthalmology and Otorhinolaryngology, School of Medicine, Universidad Complutense de Madrid, Madrid, Spain; ^2^Université de Paris, Institut National de la Recherche Médicale, U1016, Centre National de la Recherche Scientifique, UMR8104, Paris, France; ^3^Instituto de Investigaciones Biomédicas, Universidad Nacional Autónoma de México, Mexico City, Mexico

**Keywords:** phagocytosis, phagosome, CR3, necrotic debris, efferocytosis, macrophages, microglia, opsonins

Phagocytosis is a conserved cellular process for ingesting and eliminating large (≥0.5 μm) particles, including microorganisms, foreign substances, and apoptotic cells. Phagocytosis is performed by many cell types and it constitutes an essential process for tissue homeostasis. It was long considered that only specialized cells, and in particular cells of the immune system, termed professional phagocytes (macrophages, neutrophils, monocytes, dendritic cells, microglia, osteoclasts), accomplish phagocytosis with high efficiency. Other cells, such as retinal pigment epithelial cells or hepatocytes, however, are also potent phagocytes. This Research Topic provides a timely overview of the biological importance of phagocytosis for immune-related functions and for tissue homeostasis, with focus on phagocytosis of apoptotic bodies and necrotic debris that could activate undesirable inflammatory responses. This article collection goes through the latest insights on the mechanisms controlling phagocytic receptor activation, particle engulfment, and phagosome maturation. The review by Uribe-Querol and Rosales provides a bird's-eye view of multiple aspects of the process of phagocytosis. It describes the types of phagocytosis receptors known today and the phases involved in phagocytosis, including (i) detection of the particle to be ingested, (ii) activation of the internalization process, (iii) formation of a specialized vacuole called phagosome, and (iv) maturation of the phagosome to transform it into a phagolysosome. Once the process of phagocytosis is activated, the phagocyte requires profound reorganization of its cell morphology around the target in a controlled manner. This process is limited by biophysical constraints involving the receptors, the membrane, and the actin cytoskeleton. The article by Jaumouillé and Waterman discusses the major physical constraints involved in the particle internalization resulting in the formation of a phagosome and provides an extensive review of the underlying molecular mechanisms coping with these constraints. They focus on the two most-studied types phagocytic receptors, the Fcγ receptors and the complement receptor 3 (α_M_β_2_ integrin). They revise the importance of receptor accessibility and diffusion at the membrane, the protrusive forces generated by actin polymerization and describe a molecular clutch for the phagocytic integrin α_M_β_2_ involved in mechanosensing. Integrin phagocytic receptors initiate phagocytosis by a process that seems, at least initially, different from antibody-mediated phagocytosis. The review by Torres-Gomez et al. describes the phagocytic integrins and the molecular events controlling integrin activation and the downstream signaling driving particle engulfment. Authors focus on complement receptor (CR) 3/integrin α_M_β_2_, and on CR4/integrin α_X_β_2_. In addition, they briefly mention other integrins that do not bind complement, but that also function as phagocytic receptors, such as α_V_β_5_ and α_V_β_3_ integrins.

Phagocytosis is more efficient when foreign particles are labeled for phagocytosis by opsonins, which are host-derived proteins that bind specific receptors on phagocytic cells. Important opsonins promoting efficient phagocytosis include antibody (IgG) molecules and complement components. Other opsonins are not so well-known. In the article by Cockram et al. two novel opsonins for bacteria calreticulin and galectin-3, are described. Microglia, the brain-resident macrophages, release calreticulin and galectin-3 when activated by bacterial lipopolysaccharide. Both lectins can bind lipopolysaccharide on the surface of bacteria and are also recognized by the phagocytic receptors LRP1 and MerTK, respectively. These findings provide insight on how this innate immune response of microglia may promote clearance of bacteria in the brain.

Foreign particles can also be recognized directly by phagocytes. This is accomplished by non-opsonic receptors that directly bind pathogen-associated molecular patters (PAMPs) and can induce phagocytosis. Members of the family of C-type lectin receptors, including Dectin-1 (dendritic cell-associated C-type lectin-1), Mincle (macrophage-inducible C-type lectin), and DC-SIGN (dendritic cell-specific ICAM-3-grabbing non-integrin), as well as the mannose receptor have been described as important phagocytic receptors. In the review by Bonsignore et al., the carcinoembryonic antigen-related cell adhesion molecule 3 (CEACAM3) is presented as a novel and important non-opsonic phagocytic receptor of highly specialized, host-restricted bacteria. Authors also discuss the importance of CEACAM3 polymorphisms for human innate immunity against bacteria through phagocytosis.

After the particle has been internalized in a phagosome, this novel organelle suffers dramatic changes both in its membrane composition and in its contents by fusing with other intracellular vesicles. This process known as phagosome maturation, can be determined by phosphoinositide (PI) lipids, which are pivotal determinants of organelle identity, membrane dynamics, and vesicle trafficking. Yet, some microbial pathogens can modify the phosphoinositide pattern in phagocytic cells for their own benefit. The article by Leoni Swart and Hilbi describes how *Legionella pneumophila*, the causative agent of a severe pneumonia called Legionnaires' disease, alters the phosphoinositide pattern of the phagosome to create a new vesicle known as the *Legionella*-containing vacuole (LCV), where the bacterium can live and replicate inside lung macrophages. The authors summarize strategies, by which *L. pneumophila* subverts macrophage phosphoinositide lipids, particularly the important conversion from phosphatidylinositol (3) phosphate to phosphatidylinositol (4) phosphate, to promote LCV formation and intracellular replication. As mentioned, another important aspect of phagosome maturation is the change of its contents into a potent microbicidal environment. Acidification the phagosome is essential for achieving activation of many microbicidal enzymes in the phagosome. The article by Yoon et al. presents a novel mechanism by which macrophages regulate phagosome acidification upon exposure to Gram-negative bacteria. Evidence is provided on how the thioredoxin-interacting protein (TXNIP)-associated inflammasome plays a role in pH modulation through the activated caspase-1-mediated inhibition of NADPH oxidase. This acidification pathway is relevant for controlling bacterial clearance by macrophages.

Concomitant with phagocytosis, primed phagocytes perform additional functions that contribute to eliminate the ingested pathogen and to regulate inflammation. The article by Acharya et al. describes that murine macrophages increase their levels of tumor necrosis alpha (TNF-α), interleukin (IL)-1β, IL-6, and matrix metalloproteinase 9 (MMP-9) during complement-mediated phagocytosis. Authors also provide evidence that this upregulation of proinflammatory mediators downstream of CRs may be dependent on calpain-mediated inhibition of IκBα and NF-κB activation. Another cell response associated to phagocytosis is the generation of extracellular vesicles with antibacterial properties (aEV). The article by Lörincz et al. describes that complement receptor CR3 is decisive for both processes in human neutrophils. By using bone marrow derived neutrophils from genetically modified mice, they confirm that CR3 is involved in both processes. Although this receptor plays a critical role in both responses, authors also present evidence for phagocytosis and biogenesis of antibacterial extracellular vesicles to be independent processes regulated by different signaling pathways.

Phagocytosis is not only an important process for eliminating microorganisms but also for eliminating damaged and apoptotic cells. In this manner phagocytosis contributes to maintain tissue homeostasis. Detection of apoptotic cells requires particular receptors for molecules that only appear on the membrane of dying cells. These molecules include lysophosphatidylcholine, and phosphatidyl serine (PS) and they deliver to phagocytes an “eat me” signal. Because some normal cells, including activated B and T cells, may express significant levels of PS on their surface, other molecules, for example CD47, function as “don't eat me” signals. Recognition and elimination of apoptotic cells by macrophages, a process known as efferocytosis, is also essential for the resolution of inflammation. The review by Kourtzelis et al., presents our current understanding about the mechanisms regulating macrophage efferocytosis during resolution of inflammation. In special tissues, such as the brain, phagocytosis also plays an essential role in eliminating dead cells and protein aggregates. A particular type of efficient phagocytes, the microglia, is responsible for this function. In the review by Márquez-Ropero et al., the differences in the origin, lineage and population maintenance of microglia and macrophages is presented. They discuss the principles that govern efferocytosis, and describe the epigenetic, transcriptional, and metabolic rewiring by microglia and its implication in trained immunity and brain function.

Most of the studies on the clearance of cellular debris have been strongly biased toward the removal of apoptotic bodies. As a result, the mechanisms underlying the removal of necrotic cells have remained relatively unexplored. In the review by Westman et al., our emergent understanding of the phagocytosis of necrotic debris is presented. They explain the process from recognition of necrotic cells to their internalization and disposal by phagocytes. The review describes the classes of “find me” and “eat me” signals presented by necrotic cells and their cognate receptors in phagocytes, which in most cases differ from the extensively characterized counterparts in apoptotic cell phagocytosis. An example of the importance of phagocytosis of necrotic and cell debris is found in the disease retinitis pigmentosa, characterized by progressive loss of retinal photoreceptors, resulting in blindness. A special form of this disease is caused by mutations in the clearance phagocytic receptor Mer tyrosine kinase (MerTK) and the failure of retinal pigment epithelial cells to eliminate photoreceptor outer segment debris during diurnal phagocytosis. Now, a novel role for microglia in the onset of retinitis pigmentosa is presented in the paper by Lew et al.. They describe how loss of the phagocytic receptor MerTK causes microglia activation and relocalization in the retina, and that microglia activities accelerate loss of photoreceptors. These findings suggest that therapies targeting microglia may slow down the development of this blinding disease. Another example of phagocytosis eliminating necrotic cells and facilitating uptake of protein antigens is presented in the article by Murshid et al.. They describe how cells undergoing necrosis can release heat shock proteins (HSP) a highly abundant class of molecular chaperones that when released can couple to client binding proteins in the extracellular milieu. HSP can be recognized through the scavenger receptors LOX-1 (class E member oxidized low-density lipoprotein receptor-1) and SCARF1 (scavenger receptor class F member 1), expressed by dendritic cells (DC) and macrophages activating inflammatory pathways and innate immunity. Additionally, these receptors facilitate uptake of HSP and coupled antigens, and their delivery to the proteasome, leading to antigen processing, cross presentation, and stimulation of adaptive immunity.

Elimination of altered, but not dying cells, is also an essential aspect of tissue homeostasis. Several cancer cells can be recognized and eliminated through phagocytosis. Yet, some tumor cells can also block this response by expressing the anti-phagocytic (don't eat me") CD47 molecule. In the case of the non-small-cell lung cancer (NSCLC) it has been observed that some tumors develop tyrosine kinase inhibitor (TKI) resistance in patients with EGFR-mutant-mediated NSCLC. In the paper by Nigro et al., a transcriptomic analysis of NSCLC cell lines revealed selective overexpression of CD47 in patients carrying EGFR mutations. Also, CD47 expression became up-regulated following *in vitro* TKI resistance development. By inhibiting the CD47 protein using a specific monoclonal antibody, the clearance of EGFR-TKI resistant cells by phagocytes is increased. Thus, this report supports that some tumors enhance CD47 expression to avoid phagocytosis, and that CD47 neutralization by specific monoclonal antibody may be a promising immunotherapeutic option for resistant EGFR-mutant NSCLC. An interesting example of elimination of foreign cells through phagocytosis is also presented in the paper by Gavin et al.. A promising tool in the treatment of chronic inflammatory diseases is the administration of mesenchymal stromal cell (MSC). The outcomes of this therapy have been ascribed to the capacity of MSC to release a large variety of immune-modulatory factors. However, after administration most of the infused MSC are undetectable in the circulation within hours. In this report, authors found that upon contact with blood, complement proteins were deposited on MSC and complement opsonization of MSC enhanced their phagocytosis by monocytes. These findings imply that at least some of the MSC immune-modulatory effects could be mediated by monocytes that have phagocytosed them.

Phagocytosis is a complex process performed by many cell types. Much of our knowledge comes from studies of model professional phagocytes. However, one must bear in mind that not all phagocytes behave the same. An important example illustrating this notion, is presented in the article by Zajd et al.. They remind us that macrophages are a heterogeneous and plastic population of cells whose phenotype changes in response to their environment, and they explore the functional differences between peritoneal (pMAC) and bone marrow-derived macrophages (BMDM). Contrary to their hypothesis, authors found that BMDM were generally more responsive and poised to respond to their environment than pMAC. These findings are relevant for future studies, since many times these two types of phagocytes were considered to behave similarly. Finally, the review by Davies et al., describes some properties of a non-professional phagocyte, the hepatocyte, which is very important in drug detoxification and immunity. Liver function can have profound effects in metabolism, inflammation and cancer. In addition to phagocytosis/efferocytosis this review presents the latest findings on other types of endocytosis (entosis, emperipolesis, and enclysis) by hepatocytes.

Phagocytosis is a fundamental process for the ingestion and elimination of microbial pathogens and apoptotic cells. Thus, phagocytosis is vital for tissue homeostasis. In this Research Topic, we have collected a series of articles that gives us better understanding of the process of phagocytosis, however many important questions remain unsolved. For example, how different phagocytic receptors on the same cell work together? What is the role of different phagocytes in tissue homeostasis? An improved understanding of phagocytosis is essential for future therapeutics related to infections and inflammation.

## Author Contributions

EL, FN, and CR have contributed to the writing of this editorial article. All authors contributed to the article and approved the submitted version.

## Conflict of Interest

The authors declare that the research was conducted in the absence of any commercial or financial relationships that could be construed as a potential conflict of interest.

